# Cassava mosaic disease (CMD) in Benin: Incidence, severity and its whitefly abundance from field surveys in 2020

**DOI:** 10.1016/j.cropro.2022.106007

**Published:** 2022-08

**Authors:** Jerome Anani Houngue, Serge Sètondji Houédjissin, Corneille Ahanhanzo, Justin S. Pita, Mélaine S. Ella Houndénoukon, Martine Zandjanakou-Tachin

**Affiliations:** a Central Laboratory of Plant Biotechnology and Plant Breeding, Department of Genetics and Biotechnology, Faculty of Science and Technique, University of Abomey-Calavi, Benin; b Central and West African Virus Epidemiology (WAVE), Pôle Scientifique et d'innovation de Bingerville, Université Félix Houphouët-Boigny (UFHB), Bingerville, Cote d’Ivoire; c Laboratory of Molecular Plant Pathology, School of Horticulture and Green Space, National University of Agriculture, Benin

**Keywords:** CMD symptoms, Cassava mosaic virus, Virus vector, *Bemisia tabaci*, *Manihot esculenta*, CMD, cassava mosaic disease, ACMV, African cassava mosaic virus, EACMV, East African cassava mosaic virus

## Abstract

Cassava mosaic disease (CMD) is the main threat to cassava (*Manihot esculenta* Crantz) production in Benin. This study was conducted to assess CMD incidence, disease severity, and adult whitefly (*Bemisia tabaci*) populations in 11 regions of Benin. A total of 180 cassava fields across the country were assessed during June–December 2020 following the harmonized protocol of the Central and West African Virus Epidemiology program. Based on symptoms observation, CMD was present in all surveyed fields in Ouémé and Alibori regions. The highest disease incidence levels were observed in Malanville (100%), Kpomassè (86.67%), Kandi and Zagnanado (both 81.67%), Ségbanan (80%), and Avrankou (76.67%) districts. The highest mean severity scores were in Couffo (3.68), Mono (3.63), and Atlantique (3.33) regions, while the lowest was in Alibori (2.37). Adult whitefly populations (mean number/plant) were highest in Couffo (15.88) and Mono (13.00) regions and lowest in Donga (0.06). Significant relationships were found between CMD severity and whitefly abundance (*P = 0.0010*) but there was no significant relationship between whitefly numbers and CMD incidence (*P = 0.0577*). These findings indicate that CMD has expanded its range across Benin. They also provide a basis for designing a response strategy for the control of cassava virus diseases such as CMD.

## Highlights

• One of the constraints for cassava production in Benin is cassava mosaic disease.
• Cassava fields were surveyed for CMD and its vector throughout Benin.
• The disease symptoms and CMVs vectors were found in all regions of Benin.
• The incidence of CMD has reached 100% in Alibori region in northern Benin.
• CMD is still expanding in new areas of cassava production across the country.

## 1 Introduction

Cassava (*Manihot esculenta* Crantz) is one of the most important root crops in Africa as well as for millions of people in the tropics worldwide (Legg, 1999). This importance can be attributed to the ability of cassava to thrive in adverse climatic conditions and poor soils (Ntawuruhunga et al., 2007), thus making it an ideal food security crop in the tropics. It is the second most commonly crop grown after maize in Benin but it is the most important plant in multi-crop systems. It is found in a wide range of markets and provides a stable source of income and food for many households (Houngue et al., 2018). The average cassava yield in Benin is 14 t/ha (FAOSTAT, 2021), which is lower than the estimated potential of 80 t/ha (FAO, 2013). This lower yield may be due to abiotic and biotic factors that affect cassava cultivation including cassava diseases.

Cassava diseases are an important factor as they prevail even with Benin's acceptable agricultural biotic conditions. Several diseases hamper cassava production, but the most important ones are virus-related; in Benin, cassava mosaic disease (CMD) is the most serious (Sseruwagi et al., 2004). This disease of cassava is caused by cassava mosaic begomoviruses (genus *Begomovirus*, family *Geminiviridae*). It is transmitted by whitefly (*Bemisia tabaci*) and perpetuated through infected cuttings, which is the usual crop propagation method (Fauquet et al., 2005). To date, 11 species of cassava mosaic viruses have been described and cause disease in Africa and the Indian sub-continent (Patil and Fauquet, 2009; Legg et al., 2015; Fondong, 2017). Cassava viruses may cause 20–95% yield losses; their effects are more severe when plants are infected in early growth stages than in later stages (Torkpo et al., 2018). The disease is manifested by leaf mosaic patterns, leaf reduction, and leaf chlorosis, followed by plant dieback, thereby causing important economic losses for farmers (Thresh et al., 1997). Cassava viruses exhibit diverse infection dynamics such as symptom expression, progression, recovery, severity, and host range (Bull et al., 2007; Patil and Fauquet, 2009); symptoms can vary among leaves, shoots, and plants within the same cassava variety. This variation in symptoms may depend on virus strain, virus species, host plant susceptibility, plant age, and environmental factors, such as soil fertility and soil moisture availability (Hillocks and Thresh, 2000). Furthermore, high disease incidence is closely related to the use of infected cuttings and density of whitefly populations in fields (Houngue et al., 2019b), whereas high severity may be due to a high concentration of virus in plants (Tsai et al., 2022). Previous studies on CMD have been limited by the fact that they were conducted within the borders of a single country raising issues related to small sample sizes, incomplete/missing data, inadequate methods to collect/store/process data, inadequate statistical methods selected to analyze the data among others. Central and West African Virus Epidemiology (WAVE) program have attempted to address these limitations by harmonizing a data collection protocol across 10 Central and West African countries. Thus, the data was collected and stored-edge database that contains the most comprehensive set of data on CMD, allowing for more thorough investigations and development of risk mitigation strategies. High disease incidence, disease severity, and abundant whitefly populations in farmers' fields throughout Benin were found in 2015 and 2017 together with indications of the viruses possibly present (unpublished data). With the potential risk of contamination and propagation of the redoubtable EACMV-UG (East African cassava mosaic virus-Uganda) to new areas of cassava production, there was a need for an extensive survey to assess the current status of CMD in Benin. The aim of this survey was first to provide an updated status of CMD in Benin including incidence, symptom severity, and infection type; and second to assess adult whitefly populations in cassava fields in Benin.

## 2 Materials and methods

### 2.1 Survey locations

**Fig. 1:**
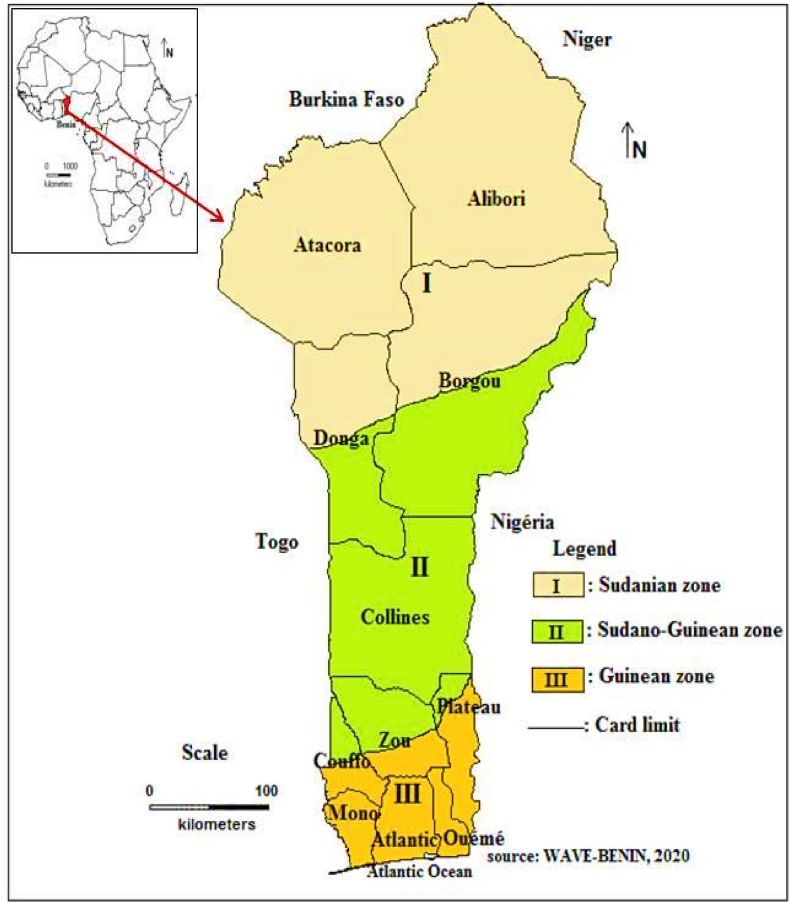
Climatic zones of Benin.

The study was carried out in 72 districts across 11 cassava-producing regions in Benin (Fig. 1). These regions have a rainfall range of 700–1500 mm per year and their soils are suitable for cassava cultivation. These regions are within three climatic zones, the Sudanian (I), Sudano-Guinean (II), and Guinean (III) zones (Fig. 1):

- Zone I is characterized by 950–1300 mm of annual rainfall with one rainy season and average temperature of 17–33 °C and relative humidity of 20–47%;
- Zone II is characterized by 1200 mm of annual rainfall with two rainy and dry seasons. The temperature average is 22.5–35.2 °C and relative humidity is 30–83%;
- Zone III is characterized by 950–1500 mm annual rainfall with two rainy and dry seasons. Annual average temperature is 24–31 °C and relative humidity is 58–95% (Afloukou et al., 2020).

Seven of the regions surveyed are major cassava-producing regions: Mono, Couffo, Atlantique, Ouémé, Plateau, Zou, and Collines.

### 2.2 Survey method

Using the harmonized WAVE sampling protocol, 72 districts in 11 regions, and a total of 180 fields were surveyed in Benin between June and December 2020 (Table 1). The surveyed fields were approximately 10 km apart, depending on the availability of fields on the accessible roads. Thirty cassava plants aged 3–6 months were randomly examined along two diagonals in an “X” pattern in each field and 15 plants were selected along each diagonal.

**Table 1:** Characteristics of regions and districts surveyed.

Regions	Surveyed districts	Geographical location	Climate zones	Major crops
Alibori	Malanville, Kandi, Ségbanan, Banikouara, Gogounou	North-Est	I	Sorghum, maize, rice, cotton
Atacora	Kérou, Pehounco, Kouandé, Natitingou, Toukountouna, Tanguiéta, Matéri, Boukoumbé, Cobly	North-west	I	Sorghum, maize, yam, cotton, cassava
Atlantique	Kpomassè, Ouidah, Toffo, Allada, Zè, Abomey-Calavi, Torri-Bossito,	South-central	III	Pineapple, maize, tomato, cassava
Borgou	Tchaourou, Parakou, N'dali, Bemberèkè, Pèrèrè, Nikki, Kalalé	North-Est	I and II	Cereal, yam, cassava, cotton
Collines	Dassa, Glazoué, Savè, Ouèssè, Savalou Bantè	Central	II	Cereal, cassava, yam, cotton
Couffo	Dogbo, Aplahoué, Klouékanmè, Toviclin, Djakotomey	South-Est	II and III	Maize, cassava, cowpea, cotton
Donga	Copargo, Djougou, Ouaké, Bassila	North-West	I and II	Maize, cassava, cowpea, cotton
Mono	Come, Bopa, Houéyogbé, Grang-Popo, Athiémé, Lokossa	South-Est	III	Maize, cassava, cowpea, tomato, pepper
Ouémé	Sèmè-Kpodji, Porto-Novo, Adjarra, Avrankou, Bonou, Adjohoun, Dangbo, Akpro-Missérété	South-West	III	Maize, cassava, groundnut, tomato, pepper
Plateau	Sakété, Ifangni, Adjaouèrè, Pobè, Kétou	South-West	II and III	Cassava, yam, maize, groundnut, tomato, pepper
Zou	Ouinhi, Zagnanado, Covè, Zakpota, Djidja, Abomey, Agbangnizoun, Bohicon, Zogbodomey	Central	II and III	Maize, cassava, groundnut, bean, tomato, pepper

### 2.3 Data collection and recording

Field data were recorded in accordance with the WAVE survey protocol. Four leaf samples per field were collected and stored in a herbarium press. The samples were labeled with an identifier composed of field and plant number and collection date. Cuttings were taken from each sampled plant and grown under greenhouse conditions in 1-L black polythene bags for observation of disease symptom expression.

In each field, whiteflies were counted on the five apical leaves of the plants examined. They were collected using an aspirator and stored in Eppendorf tubes containing 70% ethanol (Sseruwagi et al., 2004).

Data were recorded using a tablet and a data collection application (built in iForm 9.12.7) which was developed for the WAVE program by the University of Cambridge, UK's Epidemiological Modelling Group. The data were then uploaded from the tablet to iForm's cloud-based database and then integrated into the WAVE Cube multi-dimensional database – according to the WAVE survey protocols (Soro et al., 2021). Information recorded included cassava plant details as well as the location coordinates (latitude and longitude), and altitude of fields recorded using a global positioning system (Garmin eTrex, Summit HC).

When recording disease information, CMD severity was assessed based on a severity scale with range 1–5 as defined by the International Institute of Tropical Agriculture (IITA): 1, absence of infection; 2, mild infection; 3, moderate infection; 4, severe infection; and 5, very severe infection (IITA, 1990).

In each field, we recorded the adult whitefly population (on the top five leaves), the number of visible cassava fields nearby, and the size of the field. The CMD incidence was determined by the proportion of diseased plants expressed as a percentage of the total number of plants assessed per field. When determining severity, asymptomatic plants (score 1) were excluded from calculations to avoid the underestimation of the disease severity; thus field severity analysis used data on infected plants only (scores 2–5) (Sseruwagi et al., 2004).

Whiteflies were counted on the five top apical leaves of the 30 plants evaluated in each field, and the mean whitefly number was expressed as the total number of whiteflies in the field divided by 30.

### 2.4 Data processing

Data stored in the WAVE Cube could be visualized at plant, field, district and region levels as tables, graphs, or histograms to assist our analysis. Geographic coordinates (longitude and latitude) were used to map the geographic distribution of CMD, incidence, and severity in Benin. Disease incidence and severity maps were produced by means of Microsoft's Power BI tool and associated map-generating software, using the coordinates stored in the Cube. Means of CMD incidence and adult whitefly population were presented in tables and histograms. The means of incidence, severity, and adult whitefly numbers were compared using XLSTAT™ one-way analysis of variance. Regression analyses were run to examine the relationships between whitefly number and CMD incidence and severity.

## 3 Results

### 3.1 CMD symptoms assessment

**Fig. 2:**
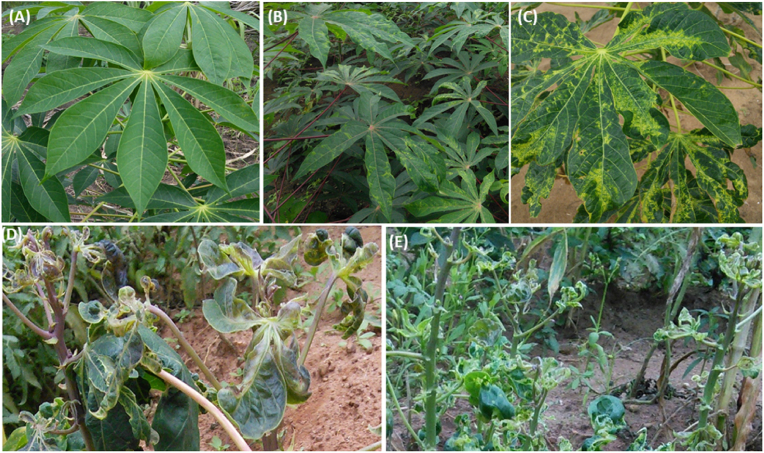
CMD symptoms in the surveyed fields: *A, asymptomatic; B, mild infection; C, moderate infection; D, severe infection; and E, very severe infection.*

CMD was observed in 145 of the 180 fields surveyed (80.55%). The disease prevalence was highest in Ouémé and Alibori regions (100%), moderate in Donga (66.67%), and lowest in Couffo (33.33%) regions (Table 2). Both mild and severe CMD symptoms were observed in the cassava fields surveyed. Symptoms comprising chlorotic blotches, leaf distortion, and mosaic were observed in eight regions: Alibori, Atacora, Atlantique, Borgou, Collines, Donga, Ouémé, and Plateau (Fig. 2).

**Table 2:** Fields showing CMD-infected fields by region.

Regions surveyed	Number of surveyed fields	Number of infected fields	Infected fields (%)
Alibori	8	8	100.00
Atacora	17	12	70.59
Atlantique	15	11	73.33
Borgou	25	22	88.00
Collines	26	21	80.77
Donga	9	6	66.67
Couffo	12	4	33.33
Mono	15	12	80.00
Ouémé	10	10	100.00
Plateau	22	21	95.45
Zou	21	18	85.71
**Total**	**180**	**145**	**80.56**

### 3.2 CMD incidence

Disease incidence differed significantly (P < 0.0001) among the 11 regions (Table 3). Disease incidence range was 9.44–74.58% among regions (Table 3). The highest disease incidence (74.58%) was found in Alibori region whereas the lowest (9.44%) was in Couffo. Moderate disease incidence levels of range 25–50% were found in seven regions (Atlantique, Borgou, Collines, Donga, Ouémé, Plateau, and Zou), while mean disease incidence of 0–25% was found in three regions (Atacora, Mono, and Couffo). Kandi and Malanville districts in Alibori region presented severe disease incidence of 81.67% and 100%, respectively (Table 3).

**Table 3:** CMD incidence in the regions and districts surveyed.

Regions	Districts	Number of fields	Number of plants evaluated	Number of CMD-infected plants	CMD incidence (%)
Alibori	Malanville	1	30	30	100.00
	Kandi	2	60	49	81.67
	Ségbanan	2	60	48	80.00
	Banikouara	2	60	45	75.00
	Gogounou	1	30	7	23.33
	*Mean incidence*				*74.58*
Atacora	Kérou	1	30	21	70.00
	Pehounco	3	90	30	33.33
	Kouandé	4	120	11	13.33
	Natitingou	2	60	8	5.00
	Toukountouna	2	60	1	1.67
	Tanguiéta	1	30	0	0.00
	Matéri	2	60	5	8.33
	Boukoumbé	1	30	1	3.33
	Cobly	1	30	19	63.33
	*Mean incidence*				*18.82*
Atlantique	Kpomassè	1	30	52	86.67
	Ouidah	3	90	19	21.11
	Toffo	2	60	8	15.00
	Allada	2	60	21	35.00
	Zè	2	60	30	50.00
	Abomey-Calavi	2	60	27	45.00
	Torri-Bossito	3	90	0	5.56
	*Mean incidence*				*30.44*
Borgou	Tchaourou	9	270	133	59.63
	Parakou	2	60	57	48.33
	N'dali	4	120	48	40.00
	Bembèrèkè	1	30	7	10.00
	Nikki	6	180	65	36.11
	Pèrèrè	1	30	0	0.00
	Kalalé	2	60	10	16.67
	*Mean incidence*				*42.80*
Collines	Dassa	4	120	51	24.29
	Glazoué	3	90	28	31.11
	Savè	4	120	31	21.67
	Ouèssè	4	120	63	51.67
	Savalou	7	210	65	30.48
	Bantè	4	120	64	54.17
	*Mean incidence*				*34.36*
Regions	Districts	Number of fields	Number of plants evaluated	Number of CMD-infected plants	CMD incidence (%)
Couffo	Dogbo	2	60	4	6.67
	Aplahoué	4	120	18	15.00
	Klouékanmè	2	60	1	1.67
	Toviclin	2	60	11	18.33
	Djakotomey	2	60	0	0.00
	*Mean incidence*				*9.44*
Donga	Copargo	2	60	0	0.00
	Djougou	2	60	11	18.33
	Ouaké	2	60	20	33.33
	Bassila	3	90	40	44.44
	*Mean incidence*				*26.30*
Mono	Come	2	60	24	40.00
	Bopa	2	60	14	23.33
	Houéyogbé	2	60	28	46.67
	Grand-Popo	3	90	2	2.22
	Athiémé	3	90	18	20.00
	Lokossa	1	30	0	0.00
	*Mean incidence*				*24.22*
Ouémé	Sèmè-Kpodji	2	60	32	53.33
	Porto-Novo	1	30	3	10.00
	Adjarra	1	30	22	73.33
	Avrankou	1	30	23	76.67
	Bonou	2	60	27	45.00
	Adjohoun	1	30	6	20.00
	Dangbo	1	30	14	46.67
	Akpro-missérété	1	30	7	23.33
	*Mean incidence*				*45.33*
Plateau	Sakété	4	120	68	56.67
	Ifangni	3	90	58	64.44
	Adja-ouèrè	2	60	10	15.00
	Pobè	3	90	16	26.67
	Kétou	9	270	98	36.67
	*Mean incidence*				*39.24*
Zou	Ouinhi	3	90	59	65.56
	Zagnanado	2	60	50	81.67
	Covè	3	90	46	51.11
	Zakpota	3	90	50	55.56
	Djidja	4	120	12	10.00
	Abomey	1	30	15	50.00
	Agbangnizoun	2	60	0	0.00
	Bohicon	1	30	9	30.00
	Zogbodomey	3	90	11	12.22
	*Mean incidence*				*35.87*
P-value		Districts			0.211
		Regions			<0.0001

**Fig. 3:**
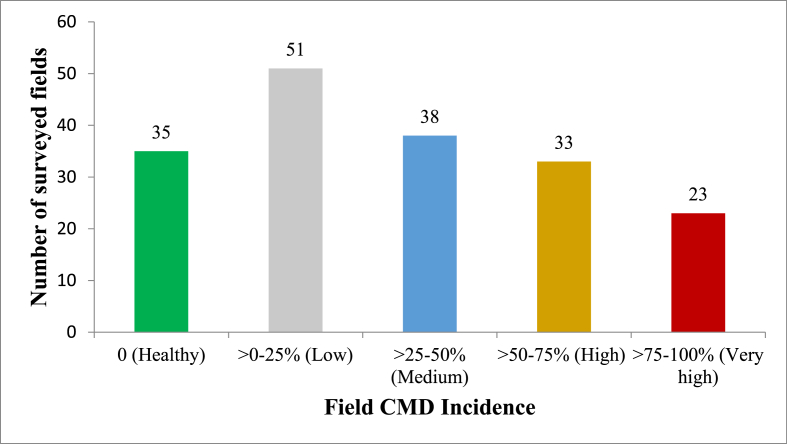
Number of fields by disease incidence level.

**Fig. 4:**
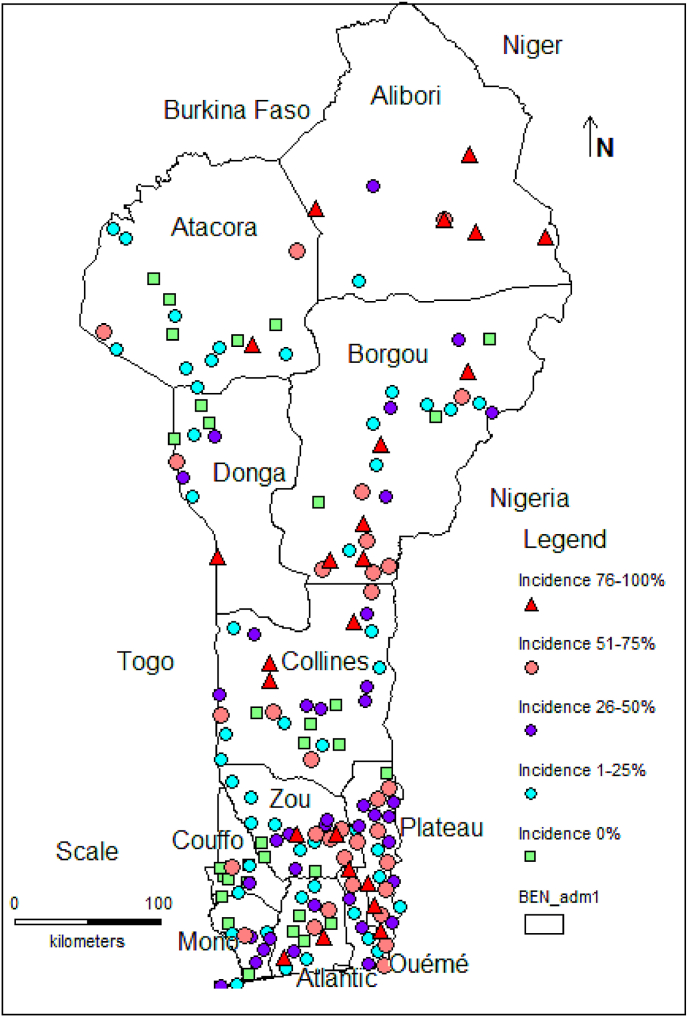
CMD incidence levels (%) by region.

We found low CMD incidence (0–25%) in 51 cassava fields, medium incidence (25–50%) in 38 fields, high incidence (50–75%) in 33 fields, and very high incidence (75–100%) in 23 fields (Fig. 3). Symptoms of CMD were found in all regions where cassava is produced in Benin. In some cassava fields, disease incidence reached 100%, e.g. Alibori region in northern Benin (see Fig. 4).

### 3.3 CMD severity

**Fig. 5:**
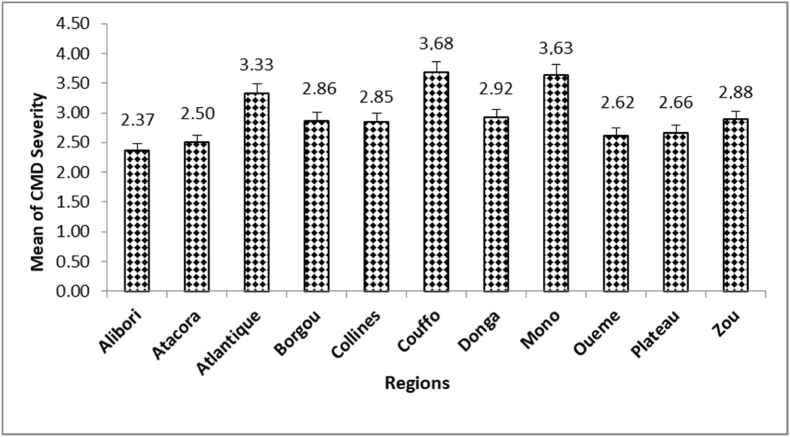
Mean of CMD severity scores (for infected plants) in regions surveyed.

Severity of CMD did not significantly differ among the regions (P = 0.1000) and among districts (P = 0.0610) (Table 4). Throughout the regions, CMD severity range was 2.37–3.68, with the severity of 3.68 in Couffo and 2.37 in Alibori region (Fig. 5). Across all plants surveyed, the overall mean CMD severity score for infected plants was 2.85.

**Table 4:** Analysis of variance of disease severity throughout the regions and districts.

Source	Degrees of freedom	Sum of squares	Average of squares	F	Pr > F
Regions	10	11.6802	1.1680	1.6363	0.1000
Districts	69	61.6214	0.8931	1.3897	0.0610

**Fig. 6:**
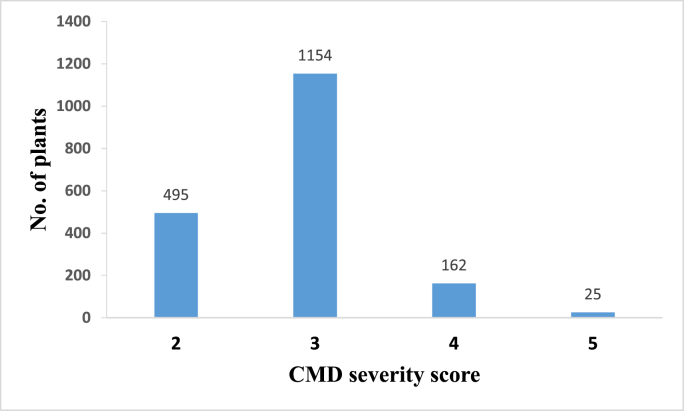
Distribution of CMD severity scores across symptomatic plants.

Of the 1836 plants that exhibited CMD symptoms in other all fields, 1154 plants were assigned a severity score of 3 whereas 495 plants were assigned a severity score of 2 (Fig. 6). The severe symptoms were seen on 187 plants, with 162 plants assigned a severity score 4 and 25 plants a severity score 5) (Fig. 6).

### 3.4 Whitefly populations, CMD severity, and CMD incidence by region

The mean numbers of whitefly on the plants that have been infested by whitefly varied significantly (P < 0.0001) 226 among regions, ranging from 0.06 (Donga region) to 15.88 (Couffo region; Table 5). With regard to the maximum number of whitefly per plant, the lowest value was found in Donga (2.00) while the largest number was observed in Ouémé (125; Table 5). In total, only 16 whiteflies were counted across 9 surveyed fields in Donga whereas 5849 whiteflies were counted across 15 surveyed fields in Mono (Table 5). By field, the lowest mean number of whitefly was observed in Donga (1.78) and the highest mean number of whitefly was found in Couffo region (476.50; Table 5).

**Table 5:** Whitefly numbers observed in surveyed fields, per region.

Region	Maximum no. of whitefly per plant	Mean no. of whitefly per plant	Minimum no. of whitefly per plant	Total no. of whitefly	No. of surveyed plants	No. of surveyed fields	Mean no. of whitefly per field (based on 30 plants/field)
Alibori	2.00	0.10 ± 0.00	0.00	23.00	240.00	8	2.88 ± 0.01
Atacora	8.00	0.09 ± 0.00	0.00	48.00	510.00	17	2.82 ± 0.01
Atlantique	28.00	2.01 ± 0.02	0.00	905.00	450.00	15	60.33 ± 3.72
Borgou	12.00	0.39 ± 0.00	0.00	290.00	750.00	25	11.60 ± 1.02
Collines	17.00	1.22 ± 0.01	0.00	954.00	780.00	26	36.69 ± 2.16
Couffo	76.00	15.88 ± 0.91	0.00	5718.00	360.00	12	476.50 ± 37.10
Donga	8.00	0.06 ± 0.00	0.00	16.00	270.00	9	1.78 ± 0.01
Mono	100.00	13.00 ± 1.21	0.00	5849.00	450.00	15	389.93 ± 34.01
Ouémé	125.00	2.74 ± 0.02	0.00	821.00	300.00	10	82.10 ± 4.23
Plateau	28.00	1.87 ± 0.01	0.00	1233.00	660.00	22	56.05 ± 3.15
Zou	24.00	1.84 ± 0.01	0.00	1160.00	630.00	21	55.24 ± 3.57
**Mean/sum**	**125.00**	**3.15**	**0.00**	**17,017.00**	**5400.00**	**180**	**77.87**

There was no proportional relationship between disease incidence and mean number of whitefly per plant. For example, a low value for mean number whitefly/plant (0.10) was associated with a high mean CMD incidence (74.58%) in Alibori region, whereas a higher mean number whitefly/plant (15.88) was associated with a lower mean CMD incidence (9.44%) in Couffo region (Table 6).

**Table 6:** Variation of CMD incidence and severity according to the mean number of whitefly per plant.

Region	Mean CMD incidence	Mean no. of whitefly per plant	Mean CMD severity
Alibori	74.58 ± 4.10	0.10 ± 0.00	2.37 ± 0.11
Atacora	18.82 ± 1.17	0.09 ± 0.00	2.50 ± 0.12
Atlantique	30.44 ± 2.21	2.01 ± 0.02	3.33 ± 0.13
Borgou	42.80 ± 3.11	0.39 ± 0.00	2.86 ± 0.09
Collines	34.36 ± 2.02	1.22 ± 0.01	2.85 ± 0.14
Couffo	9.44 ± 0.97	15.88 ± 0.91	3.68 ± 0.33
Donga	26.30 ± 1.42	0.06 ± 0.00	2.92 ± 0.12
Mono	24.22 ± 1.24	13.00 ± 1.21	3.63 ± 0.35
Ouémé	45.33 ± 3.26	2.74 ± 0.02	2.62 ± 0.14
Plateau	39.24 ± 2.96	1.87 ± 0.01	2.66 ± 0.12
Zou	35.87 ± 2.26	1.84 ± 0.01	2.88 ± 0.15
**Overall mean**	**34.00%**	**3.15**	**2.84**

The mean number of whitefly in different fields was significantly related (R^2^ = 0.7171; P = 0.0010) to CMD disease severity (Table 7), whereas there was no significant relationship (R^2^ = 0.3444; P = 0.0577) between whitefly mean per field per regions and CMD disease incidence mean per regions (Table 7). However, CMD incidence mean was significantly related (R^2^ = 0.4685; P = 0.0202) to CMD severity mean among the regions (Table 7).

**Table 7:** Relationship between whitefly mean density per field per region, CMD severity mean, and CMD incidence mean per region.

	Variables	CMD incidence – mean	Whitefly – mean	CMD severity – mean
Coefficient of determination (R^2^):	CMD incidence – mean	1		
	Whitefly – mean	0.3444	1	
	CMD severity – mean	0.4685*	0.7171*	1
Correlation matrix (Pearson	CMD incidence – mean	1		
	Whitefly – mean	−0.5869	1	
	CMD severity – mean	−0.6844	0.8468	1
p-values):	CMD incidence – mean	0		
	Whitefly – mean	0.0577	0	
	CMD severity – mean	0.0202*	0.0010*	0

Values with star (*) are different from 0 with a significance level alpha = 0.05.

## 4 Discussion

Effective management of cassava viral diseases such as CMD and cassava brown streak requires sound knowledge about these diseases and the factors that can affect their emergence. In this work, the incidence and severity of CMD were assessed in various districts of Benin, following which the relationship between this disease and whitefly abundance was established. The work consisted of surveying cassava fields throughout the territory to assess CMD prevalence and the abundance of its associated biological vector (*B. tabaci*). The survey results indicated that 145 out of 180 of these fields (i.e. 80.55%) showed CMD symptoms. This high rate of infected fields can be explained by the extensive spread of the disease in cassava production areas (Chikoti et al., 2019).

In Alibori region, all of the fields surveyed were infected. Alibori constitutes a high-risk area in which the renewal of planting material is necessary even though it is not a high cassava production area. The ease of trading cuttings within and outside the country poses a strong epidemic risk because all fields in a region could become hotspots for the virus to spread. The extensive spread of CMD can be linked to the cultural practice of farmers exchanging cuttings with one another, as well as to ignorance of the disease and the considerable damage it can cause to cassava cultivation (Chikoti et al., 2015; Houngue et al., 2018).

The moderate-to-severe severity symptoms observed in the fields may be related to the use of specific CMD-susceptible cultivars, but could also be linked to the recycle of infected cuttings (Chikoti et al., 2015), which have accumulated a high viral load. The existence of several co-infected variants can also cause severe CMD symptoms (Fondong et al., 2000).

The incidence of CMD is very high in most production areas and has reached 75–100% in the districts of Malanville, Kandi, Ségbanan, Banikouara, Zagnanado, Kpomassè, and Avrankou. Torkpo et al. (2018) also showed that the use of infected cuttings increases the CMD incidence and can lead to a considerable reduction in yield. Thus, the practice of exchanging plant material among farmers poses a serious problem for CMD management in Benin due to the potentially unlimited source of virus inoculum in the fields. This problem is exacerbated by the non-existence or inaccessibility of healthy planting material for farmers (Houngue et al., 2018).

The mean disease severity recorded in the regions could be linked to the susceptibility to CMD of cassava varieties cultivated by farmers (Houngue et al., 2019b), the existence of several virus variants in co-infections (Pita et al., 2001), viral load accumulation over several cycles, or the appearance of new severe variants (Njock and Sama, 2015; Igwe et al., 2020). The degree of susceptibility is related to the level of expression of mosaic symptoms in specific cassava varieties, as shown by Ntawuruhunga et al. (2007) in their work on Ugandan varieties TME204 and I/92/0067.

The average number (3.15) of whitefly obtained in this latest survey was not higher than that reported for 2015 and 2017 (unpublished data), but also did not exceed that reported in previous studies (4.2 in transition forest and 3.4 in dry savannah) in Benin (Legg and James, 2005). This study showed that the whitefly population was relatively low in the high-altitude regions of Borgou, Alibori, Donga, and Atacora. This observation accords with previous studies that indicated that high altitude can hamper whitefly multiplication (Cudjoe et al., 2005; Legg and James, 2005). Although previous studies (Legg and Raya, 1998) have shown a clear relationship between whitefly abundance and infection transmitted by whiteflies, such a relationship is difficult to justify due to the temporal variation of the whitefly population (Fishpool et al., 1995), and the latency time varying from 3 to 5 weeks between transmission and first symptom (Fargette et al., 1993). In our survey, we took into account the whitefly population and disease incidence variation, and found that the whitefly population in Alibori and Borgou was low whereas CMD incidence was high or medium; by contrast, we observed the opposite in southern Benin. Whitefly is not only the vector of CMD viruses, this insect also spreads other viruses (Chikoti et al., 2013). The CMD incidence is due not only to whiteflies but also to humans spreading infected cuttings. In this study, we found a significant and positive relationship between disease severity and whitefly population. This could be explained by the fact that whiteflies may transmit different variants of virus causing a co-infection which then leads to severe disease symptoms.

Cultivar response to CMD was also noted during the survey (pers. obs.). This could be linked to the substantial genetic variability that exists within cassava germplasm grown in the regions surveyed (Houngue et al., 2019a). Although virtually all of the landraces we encountered were infected by CMD, the degree of infection was variable. However, since in most cases the disease is transmitted through infected cuttings, the data collected are insufficient to draw any conclusion on the state of resistance or susceptibility of the local varieties. Therefore, these cultivars should be evaluated under a known inoculum pressure to determine the relative levels of resistance or susceptibility as shown by Houngue et al. (2019b).

In most fields, cassava is grown in combination with other crops such as sweet potato, maize, okra, yam, chili, and tomato as well as weeds (the latter have been observed to harbor whitefly). This intercropping cultivation could also affect disease severity because these other species could serve as alternative host plants for vectors of the virus (Bellotti and Arias, 2001). We suggest that a monoculture of cassava would be advantageous as this might make it possible to keep the fields clear of weeds and other crops that might harbor the viruses that cause CMD.

## 5 Conclusion

This study established that CMD is widespread in all regions of Benin and that it continues to spread to new cassava-growing areas of the country. The prevalence of the disease reached 100% in some districts. Plants showing very severe mosaic symptoms were observed in most of the growers’ fields, leading to the risk of high yield losses. The incidence and high severity of CMD recorded during the survey are also of concern. It is therefore necessary that awareness-raising and training campaigns are also carried out by agricultural extension agents, non-governmental organizations, and research institutions on the use of healthy planting material and the adoption of varieties or resistant cultivars. We propose that future research efforts should be aimed at the characterization of begomoviruses associated with CMD in Benin in order to gain knowledge on the existing virus strains prevalent in the country.

## Data availability statement

The data that support the findings of this study are available from the corresponding author upon request.

## Authors’ contributions

JSP and CA designed the study; JAH, SSH, and MSEH conducted the work; CA and MZ supervised the research; SSH analyzed the data; JAH wrote the manuscript; JAH and MZ reviewed the article; all authors read, corrected and approved the manuscript.

## Ethical approval

This article does not contain any studies with human participants or animals performed by any of the authors.

## Declaration of competing interest

The authors declare that they have no conflict of interest in this publication.
